# Effects of aging and macrophages on mice stem Leydig cell proliferation and differentiation *in vitro*

**DOI:** 10.3389/fendo.2023.1139281

**Published:** 2023-03-27

**Authors:** Jingjing Shao, Jiexia Wang, Xin Wen, Jiajia Xie, Fu Huang, Xiaoju Guan, Xinrui Hao, Ping Duan, Congde Chen, Haolin Chen

**Affiliations:** ^1^ Zhejiang Provincial Key Laboratory of Anesthesiology, Department of Anesthesiology, The Second Affiliated Hospital and Yuying Children’s Hospital of Wenzhou Medical University, Wenzhou, Zhejiang, China; ^2^ Key Laboratory of Children Genitourinary Diseases of Wenzhou City, Department of Pediatric Urology, The Second Affiliated Hospital and Yuying Children’s Hospital of Wenzhou Medical University, Wenzhou, Zhejiang, China; ^3^ Department of Gynecology and Obstetrics, The Second Affiliated Hospital and Yuying Children’s Hospital of Wenzhou Medical University, Wenzhou, Zhejiang, China; ^4^ Department of Pharmacology, The Second Affiliated Hospital and Yuying Children’s Hospital of Wenzhou Medical University, Wenzhou, Zhejiang, China

**Keywords:** stem Leydig cells, macrophages, aging, CD51, testis, testosterone

## Abstract

**Background:**

Testosterone plays a critical role in maintaining reproductive functions and well-beings of the males. Adult testicular Leydig cells (LCs) produce testosterone and are generated from stem Leydig cells (SLCs) during puberty through adulthood. In addition, macrophages are critical in the SLC regulatory niche for normal testicular function. Age-related reduction in serum testosterone contributes to a number of metabolic and quality-of-life changes in males, as well as age-related changes in immunological functions. How aging and testicular macrophages may affect SLC function is still unclear.

**Methods:**

SLCs and macrophages were purified from adult and aged mice *via* FACS using CD51 as a marker protein. The sorted cells were first characterized and then co-cultured *in vitro* to examine how aging and macrophages may affect SLC proliferation and differentiation. To elucidate specific aging effects on both cell types, co-culture of sorted SLCs and macrophages were also carried out across two ages.

**Results:**

CD51+ (weakly positive) and CD51++ (strongly positive) cells expressed typical SLC and macrophage markers, respectively. However, with aging, both cell types increased expression of multiple cytokine genes, such as IL-1b, IL-6 and IL-8. Moreover, old CD51+ SLCs reduced their proliferation and differentiation, with a more significant reduction in differentiation (2X) than proliferation (30%). Age matched CD51++ macrophages inhibited CD51+ SLC development, with a more significant reduction in old cells (60%) than young (40%). Crossed-age co-culture experiments indicated that the age of CD51+ SLCs plays a more significant role in determining age-related inhibitory effects. In LC lineage formation, CD51+ SLC had both reduced LC lineage markers and increased myoid cell lineage markers, suggesting an age-related lineage shift for SLCs.

**Conclusion:**

The results suggest that aging affected both SLC function and their regulatory niche cell, macrophages.

## 1 Introduction

Testosterone (T) produced by mammalian testicular Leydig cells (LCs) plays a critical role in the maintenance of the reproductive function and general well-being of males. Serum T decreases with age, which may trigger a number of metabolic and quality-of-life changes, including decreased lean body mass, bone mineral density, muscle mass, and strength and increased obesity and cardiovascular problems (1, 2). Although the mechanism by which serum T decreases in aging males is still not completely clear, changes in LC steroidogenic function and/or numbers may be involved. In adults, stem cells play a critical role in maintaining tissue homoeostasis by replacing ost cells. The activity of stem Leydig cells (SLCs) and the maintenance of LC homoeostasis by SLCs, in both young and aged adults, are not well studied.

There are different hypotheses about the mechanisms by which cells age. In general, these hypotheses can be classified into two major categories: intrinsic and extrinsic. The intrinsic hypothesis implies that it is what takes place inside the cells that cause cells to age, while the extrinsic hypothesis implies that exogenous factors from the environment induce (accelerate) cell aging. Intrinsic causes include telomere loss, redox homeostasis and free radical damage, genetic instability induced by DNA damage, DNA methylation, mitochondrial DNA damage, and abnormal protein accumulation caused by dysfunctional autophagy and protein quality control systems (3, 4). Extrinsic causes include a mixture of paracrine factors collectively known as the senescence-associated secretory phenotype (SASP) (5), which includes growth factors, chemokines, proinflammatory cytokines and matrix proteases. The age-related changes in both intrinsic and extrinsic effects on SLC function are not well understood.

In rodents and human, adult LCs develop from SLCs during puberty (6, 7). Such differentiation activity is maintained through adulthood into old age, since elimination of LCs by ethane dimethane sulfonate (EDS) from adult or aged rat testes resulted in full regenerations of functional LC population in 8 weeks (8, 9). SLCs were reported in the peritubular and perivascular locations in the testis and express a series of specific marker proteins, including PDGFRα (7, 10), nestin (11, 12), NR2F2 (13, 14), CD51 (12, 15, 16), P75NTR (12, 17), CD90 (18), GLI1 (19), TCF21 (20), and CD248 (21). SLCs isolated using most of these markers were able to generate new LCs when they were transplanted into peer testes whose adult LC populations were partially or completely eliminated by EDS (12, 15, 17, 21). The previous studies showed that one particular marker CD51, an integrin, could be used to identify and FACS isolate SLCs from a testicular cell suspension (12, 16). However, FACS generated not one population of CD51-positive cells but two. Our initial characterization of the two populations showed that one population also expressed SLC markers and the other macrophage markers.

The aim of the present study is to examine the effect of both aging and testicular macrophages on SLC function and how aging may affect the interaction between the two cell types. Taking advantage of the two populations of CD51-positive cells, we were able to isolate the two cell types from the same cell suspension by their differential expression of CD51 (16). In the current study, we used the same protocol to isolate CD51-positive SLCs and macrophages from both young and old mouse testes and compared their proliferation and differentiation potential *in vitro*. The results indicate that both aging and the presence of macrophages significantly affected the proliferation and differentiation of SLCs.

## 2 Materials and methods

### 2.1 Chemicals

DMEM/F12 culture medium, BSA, ITS, 2-mercaptoethanol, LIF (Leukemia Inhibitory Factor), dexamethasone, d3-T and penicillin/streptomycin were from Sigma-Aldrich (St. Louis, MO). B27, N-2 Supplement, non-essential amino acids, fetal bovine serum (FBS), and collagenase type IV were from Gibco (Carlsbad, CA). PDGFBB, oncostatin-M, EGF and FGF were from Pepro Tech (Rocky Hill, NJ). Chicken embryo extract was from US Biological (Salem, MA). SAG was from Cayman (Ann Arbor, MI). Human LH was from MyBioSource (San Diego, CA).

CD51-PE antibody was from eBioscience (San Diego, CA). F4/80-APC was from MultiSciences (Hangzhou, CHN). CYP17A1 antibody was from CST (Beverly, MA). CD51 and F4/80 antibodies were from Abcam (Cambridge, UK). The detailed information about the manufactures and dilution of antibodies can be found in **Supplementary Table S1**. The primers of qPCR are summarized in **Supplementary Table S2**.

### 2.2 Animals

Adult male C57BL/6 mice with different ages (“young” = 8 to 12-week-old and “old” = 18 to 25-month-old) were provided by the Shanghai Laboratory Animal Center (Shanghai, China). Mice were maintained in Shanghai Laboratory Animal Center under controlled light (12h light: 12h dark) and temperature (22°C), with free access to water and mice chow. Animals were brought to the animal facility of Wenzhou Medical University two weeks before the experiments began. All procedures were conducted with approval by the Wenzhou Medical University Animal Care and Use Committee and in accordance with the NIH Guide for the Care and Use of Laboratory Animals.

### 2.3 Isolation and culture of CD51 positive cells from young and aged mice

The CD51 positive cells were isolated according to a protocol published previously with minor modifications (16). In brief, the parenchyma of testes was digested with 2 mg/ml collagenase type IV in DMEM/F12 medium at 37°C for 35 min. The cell suspension was filtrated with a nylon filter (300 mesh) and centrifuged, and the pellets washed twice with D-Hank’s Balanced Salt Solution (D-HBSS). Single-cell suspensions were subsequently suspended in DMEM/F12 media with 0.5% BSA and labeled with CD51-PE antibody for 45 min at 4°C. The CD51+ (weakly-positive) and CD51++ (strongly-positive) fractions were isolated by FACS (Influx Cell Sorter, Becton Dickinson), according to the previously published protocol (16). CD51+ were previously identified as SLCs, and CD51++ were identified as macrophages (16)

The CD51+ and CD51++ cells were used for immunocytochemical staining, RNA isolation, or LC differentiation *in vitro*. CD51+ were cultured either alone or in combination with CD51++ depending on experimental design. Co-culture experiments were carried out with cell numbers seeded as a 3:1 ratio (CD51+: CD51++), based on the interstitial somatic vs immune cells of adult rodent testes (22, 23). Cells were cultured in 96-well or 48-well plates containing 1x10^4^ of CD51+ cells and/or 3x10^3^ CD51++ cells. The expanding medium (DMEM/F12) contained 2.5 ng/ml ITS, 0.5% N-2, 1% B27, 10 ng/ml oncostatin-M, 10 ng/ml PDGFBB, 10 ng/ml FGF, 10 ng/ml EGF, 0.5 nM dexamethasone, 0.5 ng/ml LIF, 2.5% chicken embryo extract, 0.05 mM β-mercaptoethanol, 0.5% non-essential amino acids, 5% FBS and 100 IU/ml-100 µg/ml penicillin-streptomycin. The cells were cultured at 35°C in a humidified incubator with 5% CO_2_. The medium was changed every three days.

### 2.4 Differentiation of CD51-positive cells

To investigate effects on T production, after reaching approximately 80% confluence, sorted cells were switched to differentiation-inducing medium (DMEM/F12, 0.1% BSA, 1X ITS, 2 ng/ml LH, 0.5 μM SAG and 5 mM lithium chloride). Medium was changed every day, and the differentiation process lasted for 4 days. The decanted medium was kept at -20°C for testosterone assays. The cells were either lysed for RNA extraction or fixed for immunofluorescent- or enzymatic-staining for steroidogenic proteins.

### 2.5 Immunofluorescence or enzymatic staining of marker proteins

Unsorted testicular cell suspensions or seminiferous tubules isolated under a dissecting microscope were suspended in DMEM/F12 medium containing 0.5% BSA. Cells or tissues were fixed with 4% paraformaldehyde for 30 min and blocked with 10% goat serum for 1 hour at 22°C. They were then incubated with CD51 and/or F4/80 (macrophages specific) antibodies overnight at 4°C. The cells and tissues were finally incubated with the fluorescence-conjugated anti-rabbit or anti-rat secondary antibodies for 1 h at room temperature. Cells were stained with DAPI before observation and photographed with a Nikon Eclipse 800 microscope.

For trypan-blue tracking macrophages *in vivo*, the adult mouse was injected intraperitoneally with saline containing 3% trypan blue and cells were collected 24 h later. Testicular cells were then isolated as described above. Cell suspensions were then stained with CD51-PE antibody for 45 min at 4°C and observed under fluorescence microscopy. The co-labeled cells were photographed under both fluorescence and bright light.

For CYP17A1 staining of differentiated SLCs, the CD51+ cells were stained with the antibody before and after the differentiation. The cells were incubated with the CYP17A1 antibody (1:200) at 4°C overnight and then with the secondary fluorescence-conjugated antibody (1:500) for 1 hour at room temperature. After washing, they were examined and photographed with a Nikon Eclipse 800 microscope. HSD3B was stained for enzymatic activity for 30 mins with a solution containing the substrate (etiocholan-3β-ol-l7-one), cofactor (NAD+), and color-revealing chemical (nitro blue tetrazolium), performed as previously reported (24).

### 2.6 Proliferation and cell number assays

Cell proliferation was assayed using an EdU labeling kit (Thermo Fisher, USA). After three days in culture, sorted cells cultured on cover-slips were incubated with EdU (10μM) for 6 h. The incorporated EdU was visualized by a Click-iT™ EdU Alexa Fluor™ 488 Imaging Kit (Thermo Fisher, USA). DNA was stained by DAPI before the cells were examined and photographed by fluorescence microscope. The EdU+ cells were quantified from 3 independent experiments by counting 7 fields (about 200μm × 200μm area) in each experiment. The EdU+ cells were expressed as a ratio of EdU+ nuclei vs total nuclei labeled by DAPI.

### 2.7 RNA extraction and qPCR

Total RNA was extracted from freshly isolated or cultured cells by RNeasy mini kit (Qiagen, USA), according to the manufacturer’s instructions. The cDNA was synthesized from total RNA by a random hexamers kit (Bio-Rad, USA). The expression of cell marker or steroidogenic genes was analyzed by qPCR. The genes analyzed included Cyp17a1, Cyp11a1, Scarb1, Lipe, Acta2, Cd146, Cd31, Cd45, Ddx4, Adgre1, Csf1r, Tnf-a, IL-6, IL-8, IL-1b, Cd51, Nes, Coup-tf2, Tcf21, Cd105, Cd73, Notch1, Notch2, Notch3, Hes1, Ptch1, Ptch2, Smo, Sufu, Lhcgr, Star, Hsd3b1, Hsd17b3, Des, Pdgfra, Pdgfrb, Acta2, Gli1, Gli2 and Gli3. The amplification was carried out with a SYBR^®^ Green PCR Master Mix Kit, using a protocol consisting of 95°C for 5 min, followed by 40 cycles of 95°C (10 s), and 60°C (30 s). A universal expression gene ribosomal protein S16 (Rps16) was used as an internal control. The expression levels were calculated using the Delta-Ct method and adjusted to *Rps16*.

### 2.8 Testosterone assay by ultra-performance liquid chromatography–mass spectrometer/mass spectrometer

Testosterone levels in the culture media were assayed by UPLC-MS/MS (XEVO TQD triple quadrupole MS, Waters Corp, US), following Acquity UPLC separation) as reported previously (19, 25). Working solutions of testosterone were diluted from stock solutions by methanol. Testosterone-free cell culture media was used to dilute the d3-testosterone internal standard (IS) working solution. The collected cell medium (50 µl) was mixed with acetonitrile (100 µl) and the IS working solution (5 µl). After vibration for 3 minutes, the mixture was centrifuged at 12,000 × g for 15 min. The supernatant (10 µl) was loaded into the UPLC-MS/MS for quantification of testosterone. All samples were run in duplicate, with an intra-assay coefficient of variation being less than 10%. For some experiments, testosterone concentrations were adjusted by the total numbers of cells estimated for each treatment to reduce the bias from cell proliferation differences.

### 2.9 Statistics

Data was expressed as the mean ± standard errors of the assays from 3-7 independent experiments. To reduce the systematic errors among the experiments, data from some of the assays were pre-normalized against the reading of the young control group (for qPCR assays) or the day 4 value (for testosterone assays) of the same experiment. One-way analysis of variance (ANOVA) was used for comparisons of multiple groups. If group differences were revealed by ANOVA (P < 0.05), differences between individual groups were determined with the SNK test, using SPSS (IBM, US) statistical software package. GraphPad Prism 8.0 (GraphPad Software Inc., US) or Excel (Microsoft Office) were utilized to draw diagrams. Statistical significance was defined at P<0.05.

## 3 Results

### 3.1 Characterization of CD51 positive cells in young adult mice

**Figure 1:**
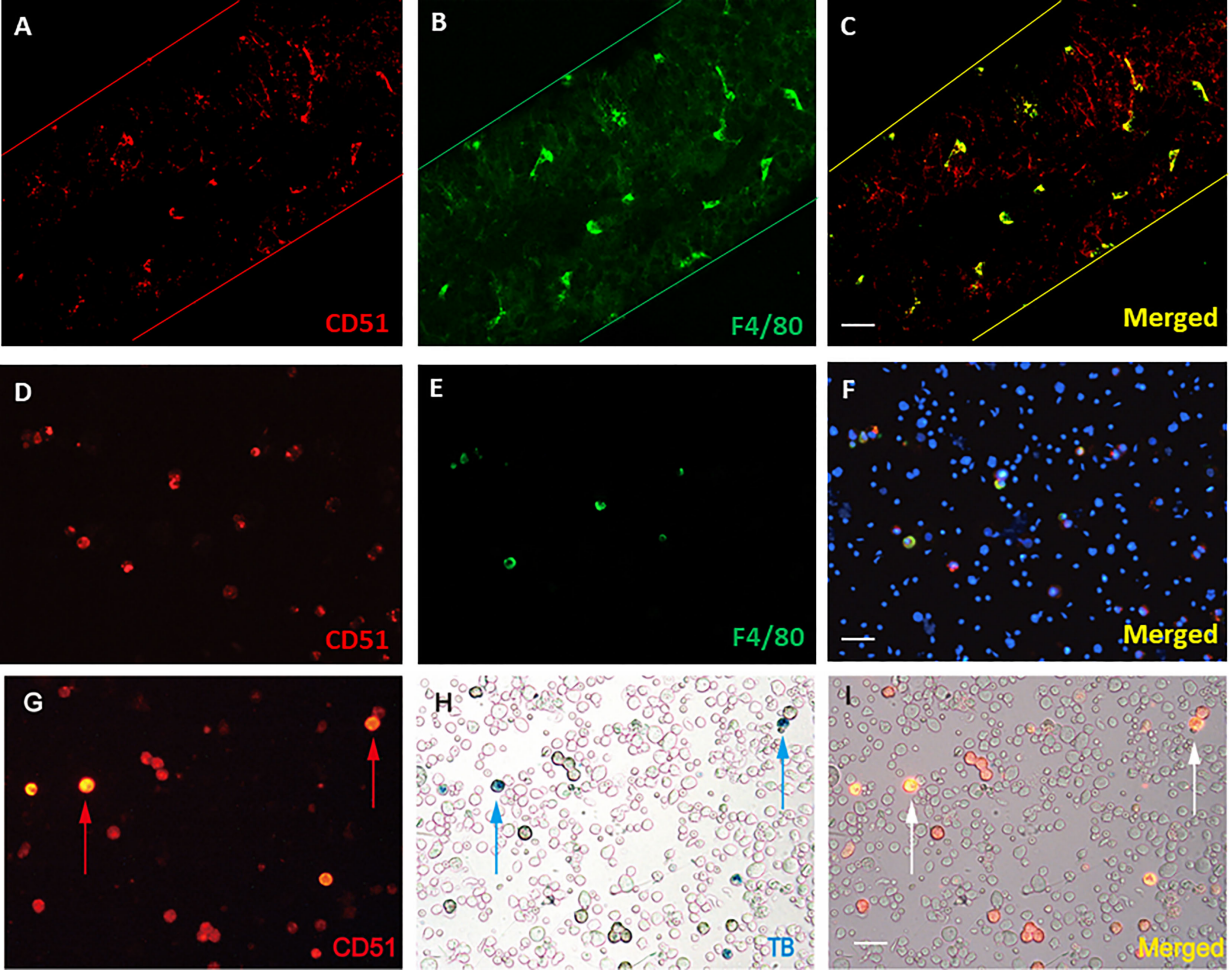
Co-localization of CD51 and macrophage marker F4/80. **(A–C)** Mounted seminiferous tubules were co-stained with CD51 and F4/80 antibodies. **(D–F)** Unsorted testicular cell suspension was co-stained with CD51 and F4/80 antibodies. **(G–I)** Macrophages labeled by Trypan blue phagocytosis (blue arrows) were positively-stained with the CD51 antibodies (red or white arrows). Scale bar: 50µm in length.

CD51 was previously shown to be a unique cell surface marker to isolate both SLC and macrophages from cell suspension from adult C57BL6 mouse testes (16). In that study, that CD51 strongly positive cells were macrophages and not SLCs was established by RNA evidence (16). To confirm that these two populations of cells exist both *in vitro* and *in vivo* using protein markers, both whole-mounted seminiferous tubules (**Figures 1A–C**) and unsorted testicular cell suspensions (**Figures 1D–F**) were co-labeled with antibodies against CD51 and macrophage marker F4/80. F4/80 consistently co-localized with CD51, but the CD51 antibody labeled more cells than F4/80, confirming that the F4/80 population is included in CD51 population. It is well-known that the live macrophages can be labeled *in vivo* by trypan blue administration based on the ability of macrophages to accumulate the dye through endocytosis (26, 27). Co-staining of unsorted testicular cells of mice administered trypan blue revealed that the trypan blue-containing cells were also positive for CD51, confirming that CD51 strongly positive cells were macrophages (**Figures 1G–I**).

### 3.2 Characterization of young and old adult CD51+ and CD51++ cells

**Figure 2:**
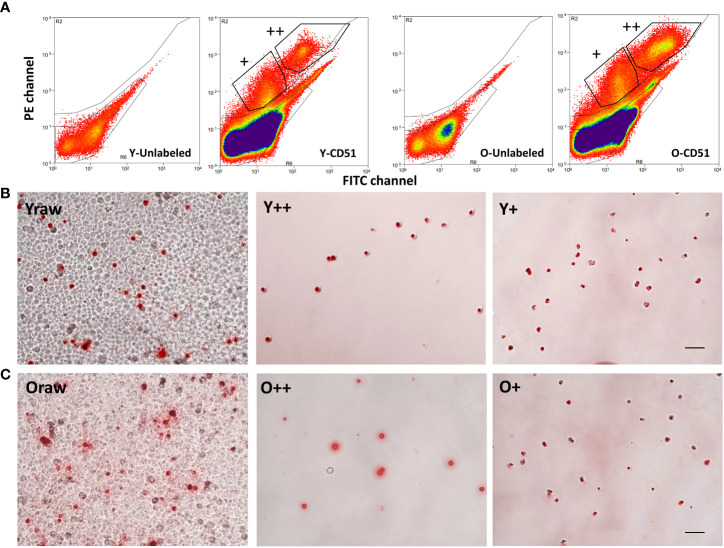
Fluorescence-activated cell sorting (FACS) of CD51-positive cells from young (Y) and old (O) testicular cell suspensions. **(A)** Testicular cell suspensions from Y or O testes without label (Y- and O-unlabeled) or with CD51-PE antibody labeled (Y- and O-CD51) were analyzed by PE and FITC channels. CD51-negative cells distributed below the diagonal line while CD51+ and CD51++ cells distributed above the diagonal line. **(B, C)** CD51-labeled Y (Yraw) and O (Oraw) testicular cells were separated into CD51 strongly positive (++) and weakly-positive (+) Y and O cells. Scale bar: 50µm in length.

To characterize CD51+ and CD51++ cell populations from both young (Y) and old (O) adult C51BL6 mice, testicular cell suspensions from both ages were tagged with CD51-PE antibody and isolated by FACS (**Figure 2**). Since testicular cells have high auto-fluorescence with broad wavelength distributions, the FITC channel (X-axis) was brought in to correct the background signal in the PE channel (Y-axis) (**Figure 2A**). For unlabeled cell preparations, all cells located along the diagonal lines, suggesting that the auto-fluorescence distributed evenly across the PE and FITC channels (**Figures 2A**, Y- and O-Unlabeled). This makes it possible to use the FITC channel readings to correct PE channel signals. For cells stained by CD51-PE antibody, two subgroups appeared on the top of the diagonal line, suggesting specific staining by CD51-PE antibody (**Figures 2A**, Y-CD51 and O-CD51). The group with the higher PE signal was considered CD51++ (strongly-positive, 0.88% ± 0.19% for Y and 2.27% ± 0.36% for O, n=4) cells, and the group with lower PE signal was considered CD51+ (weakly-positive, 1.15% ± 0.23% for Y and 1.21% ± 0.28% for O, n=4) cells. Interestingly old testes have significantly higher number of CD51++ cells than the young testes, while CD51+ cells were similar between the two ages, suggesting that old testes may have more macrophages. The isolated cells were checked again by fluorescence microscopy and found consistently to be more than 95% pure for both CD51++ and CD51+ cells of two ages (**Figures 2A, B**).

To confirm that aging specifically increased the percentage of CD51++ macrophages, testicular cell suspensions of both ages were co-stained with CD51-PE and macrophage marker F4/80-APC antibodies (**Supplementary Figures S1, S2**). The flow cytometry profiles of both ages indicated that the percentage of macrophages of the old (3.01%, total number in quadrants I and II of PE-APC plot of the old minus the cells with auto-fluorescence) was higher than the number of the young cells (1.93%), while CD51-positive SLCs were opposite (0.83% for the old vs 1.84% for the young, quadrants III of the PE-APC plot). These results confirmed that old testes contain more CD51++ macrophages than young. Also, the results confirm that cells with strong CD51-labeling co-stained with macrophage-specific F4/80 antibody, as expected.

### 3.3 Effect of aging on CD51 + and CD51++ cell gene expression

**Figure 3:**
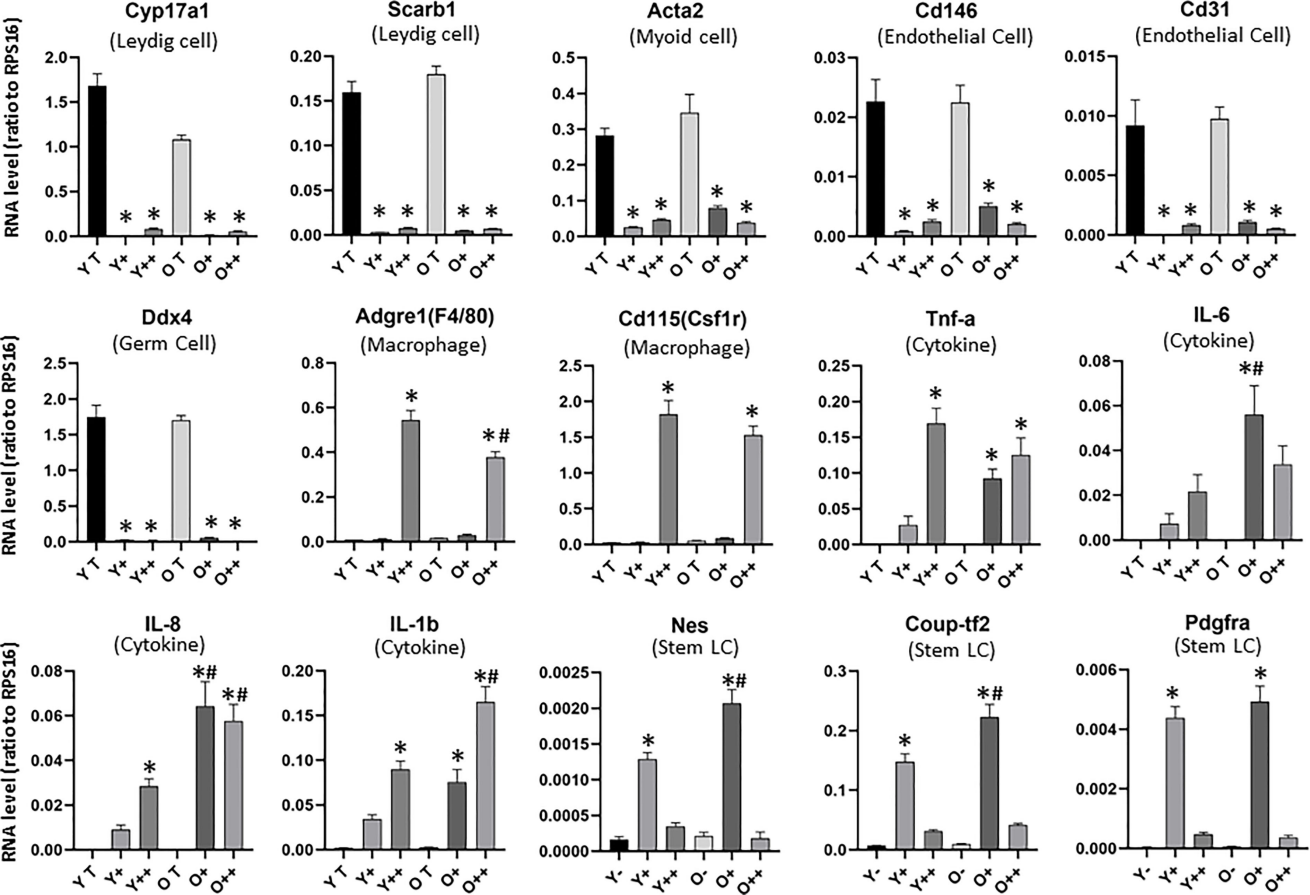
Expression of testicular cell marker genes in isolated CD51-positive cells of the young and old testes. RNAs from whole testis (YT or OT) or CD51-negitive cells (Y- or O-) were used as controls. Cell types (marker genes) include: Leydig cell (Cyp17a1 and Scarb1), Smooth muscle cells (Acta2), vascular endothelial cells (Cd146 and Cd31), germ cells (Ddx4), macrophages (Adgre1and Cd115) and stem Leydig cells (Nes, Coup-tf2, Pdgfra). A few inflammatory cytokine genes (Tnfa, IL-6, IL-8, and IL-1b) are also shown. The data are expressed as mean ± SEM of cells from three individual experiments. *^,#^Significantly different from age-matched YT/OT or Y-/O- controls (*) or from same cell type from young animals (^#^) at P < 0.05 respectively.

To more fully characterize the two CD51 positive cell types in young and old cells, qPCR was performed immediately after isolation (**Figures 3**, **S3, S4**). Both CD51 positive cell groups expressed negligible amounts of marker genes for Leydig cells (Cyp17a1, Scarb1, Cyp11a1, and Lipe), smooth muscle (myoid) cells (Acta2), blood vessel endothelial cells (Cd146 and Cd31), immune cells except macrophages (Cd45), and germ cells (Ddx4). The typical macrophage marker genes (Adgre1 and Cd115) were expressed exclusively by CD51++ cells in both age groups and confirmed their identification as macrophages. Other typical proinflammatory cytokine genes, including Tnfa, IL-6, IL-8, and IL-1b were expressed by both CD51+ and CD51++ populations for both age groups.

Interestingly, aging affected the expression of some, but not all genes for each CD51 population. For CD51++ cells, aging significantly reduced two surface marker genes Adgre1 and Cd115, while significantly increased cytokine genes IL-8 and IL-1b. Interestingly, the expression of most of the cytokine genes were also up-regulated in CD51+ cells with age. These results suggest that aging may be associated with increased inflammatory stress in the testis.

To further characterize the CD51-positive populations, we checked the expression of SLC marker genes (**Figures 3**, **S3, S4**). In addition to Cd51 (**Figure S3**), most of the potential SLC genes were almost exclusively expressed by CD51+ cells and not CD51++, including Nes, Coup-Tf2, Pdgfra (**Figure 3**), Tcf21 and Pdgfrb (**Figure S3, S4**). However, other SLC marker genes, such as CD90 and P75ntr, were not enriched from CD51 antibody sorting (**Figure S4**). In addition, other surface marker genes, which have not previously reported for SLCs, were significantly enriched in CD51+ cell, including Cd105 and Cd73 (**Figure S3**), and Des, Cd44, Cd14, and Cd34 (**Figure S4**). Since SLCs from the fetal and neonatal testis respond to Notch and DHH signaling, we also checked signaling molecules associated with the two niche factors. CD51+ cells exclusively expressed some of the Notch (Notch2, Notch3 and Hes1) and DHH (Ptch1 and Smo) signaling genes but did not enrich others, such as Notch1 and Ptch2. Interestingly, compared to CD51- cells, CD51+ cells expressed lower levels of Sufu, a negative regulator of DHH signaling.

For most of CD51+ SLCs marker genes, aging significantly up-regulated their expression, including Cd51, Nes, Coup-tf2 and Cd73 (**Figures 3**, **S3**) and Des, Pdgfrb Cd44, Cd14, and Cd34 (**Figure S4**). Aging rarely down-regulated any marker genes except Ptch1, a DHH receptor. The down-regulation of DHH receptor Ptch1 and up-regulation of Notch2 and Notch3 suggest the possibility that old SLCs may reduce their differentiation activity.

### 3.4 Effect of aging on SLC proliferation and differentiation

**Figure 4:**
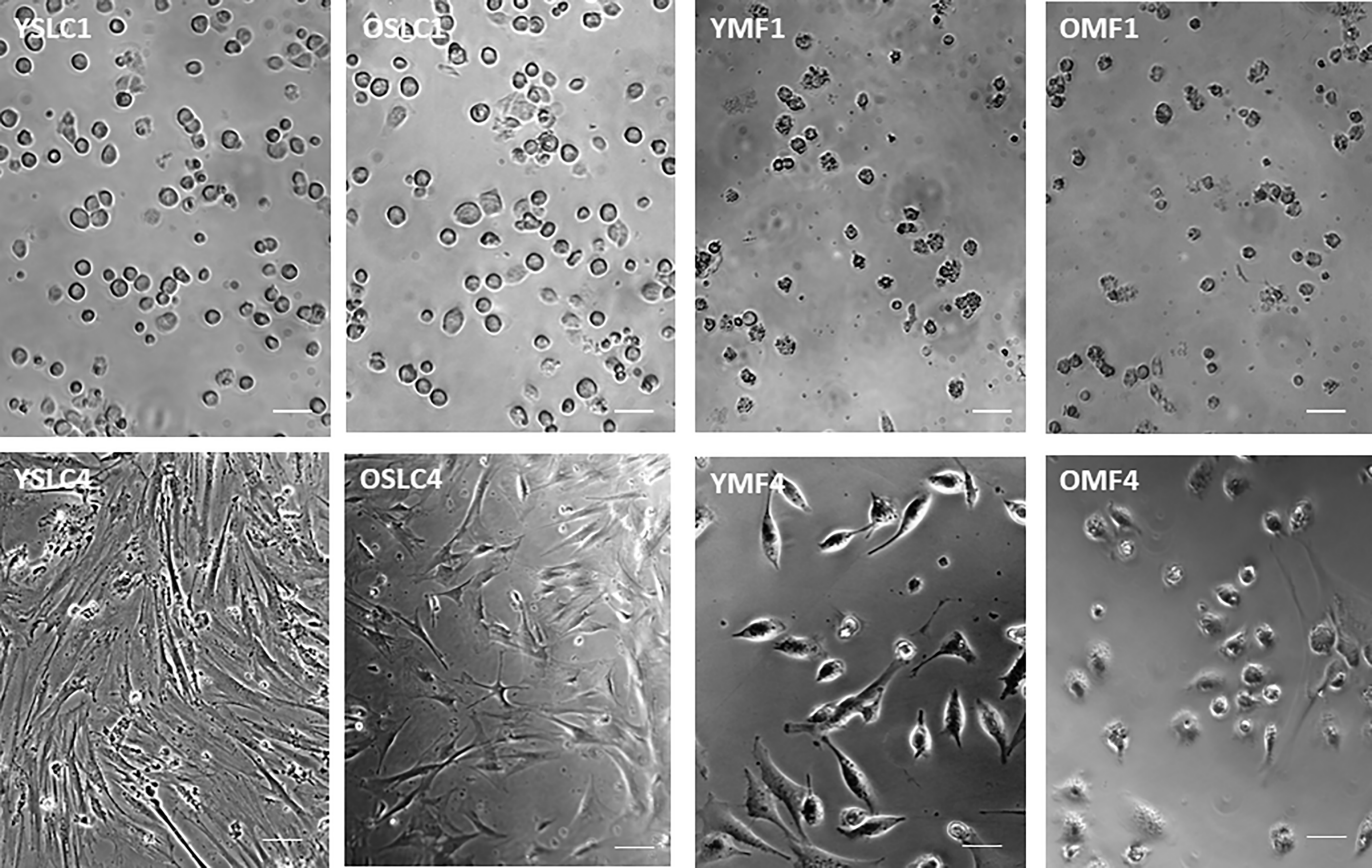
Culture of CD51+ cells (SLCs) and CD51++ cells (macrophages) from young and old testes. YSLC1 and OSLC1: Newly isolated (day 1) young and old CD51+ SLCs were round with evenly distributed cytoplasm. YMF1 and OMF1: Newly isolated (day 1) young and old CD51++macrophages were not uniform in shape and had large numbers of granules in the cytoplasm. YSLC4 and OSLC4: Young and old SLC spread out after 4 days in culture with morphology of typical mesenchymal stem cells. Also, Y cells spread better than the O cells. YMF4 and OMF4: Young and old CD51++ macrophages did not spread as well as CD51+ SLCs after 4 days in culture. Thick scale bars: 50µm in length. Thin scale bars: 20µm in length.

The freshly sorted CD51+ SLCs from both young (YSLC) and old (OSLC) testes were round and rapidly attached and spread along the culture surface (**Figure 4**, top-left, 1 day in culture). They were spread out completely by day 4, and young cells spread better than old (**Figure 4**, down-left). The morphology of SLCs was similar to that of typical mesenchymal stem cells. Freshly sorted CD51++ macrophages (YMF and OMF), however, were not uniform in shape and had large numbers of granules in the cytoplasm (**Figure 4** top-right, 1 day in culture). They were only spread out partially by day 4 in culture (**Figure 4**, bottom-right). These characters are consistent with typical macrophage cultures.

**Figure 5:**
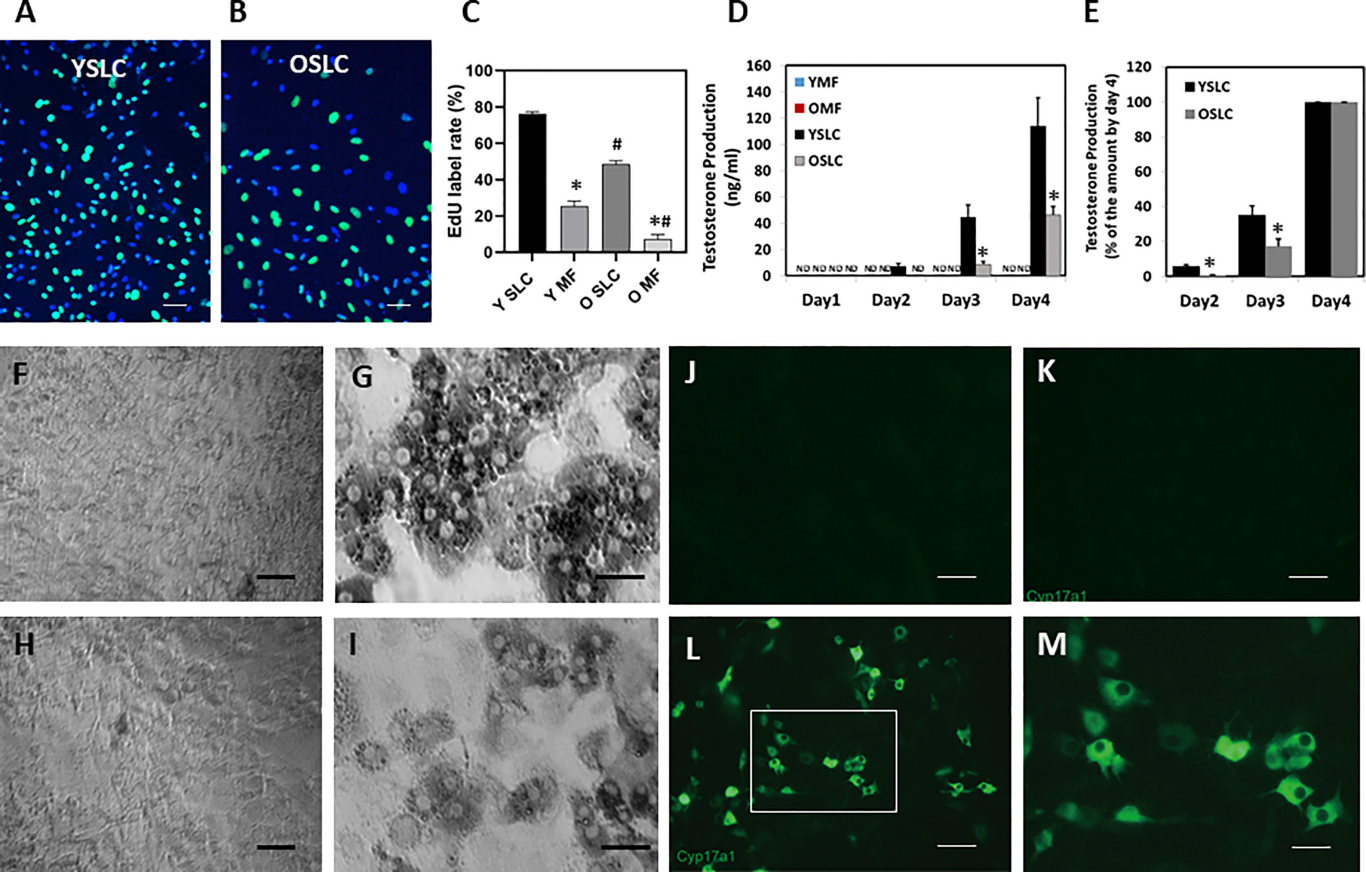
Effect of aging on the proliferation and differentiation of CD51+ SLCs and CD51++ macrophages. **(A, B)** Young and old CD51+ SLCs were labeled with EdU for 6 hours in day 3 of the culture. **(C)** The rates of EdU+ CD51+ SLCs and CD51++ macrophages were quantified. **(D)** Testosterone production of CD51+ SLC and CD51++ macrophages after induction of differentiation. **(E)** Testosterone production by differentiated CD51+ SLCs on days 2 and 3 was expressed as percentage of day-4 cells. **(F–I)** Expression of HSD3B activity by CD51++ macrophages **(F, H)** or CD51+ SLC **(G, I)** 4 days after induction of differentiation. **(F, G)** Young cells. **(H, I)** old cells. **(J–M)** Expression of CYP17A1 proteins by young CD51+ SLC after 4 days in culture. **(J)** CD51+ SLC without primary CYP17A1 antibody. **(K)** CD51+ SLC before differentiation. **(L, M)** CD51+ SLC 4 days after induction of differentiation. ND: not detected. The data are expressed as mean ± SEM of three individual experiments. *^,#^Significantly different from age-matched YSLC or OSLC controls (*) or from same cell type from young animals (^#^) at P < 0.05 respectively. Black scale bars: 50µm in length. White scale bars: 20µm in length.

To assess proliferation activity, cultured cells were labeled with EdU for 6 hours on day 4 (**Figures 5A, B**). The percentages of labeled cells were quantified (**Figure 5C**). In general, CD51++ macrophages divided slower than CD51+ SLCs; and old cells divided slower than their young counterparts. By day 4, about 80% of young CD51+ SLCs were labeled by EdU, while only 50% of old cells were the same. Similarly, old CD51++ macrophages were labeled at a significantly lower level than young.

To compare the potential of cells to develop into LCs, we switched the two populations for both ages from expanding medium into LC differentiation-inducing medium (16) after they reached about 80% confluence. No testosterone was detected in the media of either age group one day after cultures were switched to differentiation-inducing medium. However, testosterone was detected in the medium of young CD51+ SLCs, but not that of old CD61+ SLCs, by day 2 (**Figure 5D**). By day 4, young CD51+ SLCs produced 5 times more testosterone than old cells. To examine how aging may affect the differentiating efficacy at earlier time points, testosterone levels at day 2 and 3 were normalized against day 4 values for both young and old cells (**Figure 5E**). Compared to day 4, old cells produced relatively less testosterone than the young by day 2 and 3, suggesting that the age-related defect was even more significant in the early days. In contrast to CD51+ SLCs, no testosterone was detected (ND) in CD51++ macrophage culture media on any day.

To confirm that CD51+ SLCs gained steroidogenic activity upon differentiation, cells were stained for LC markers HSD3B (enzymatic activity staining, **Figures 5F–I**) or CYP17A1 (immunofluorescent staining, **Figures 5J–M**) after differentiation. Only CD51+ SLCs gained the abilities to express HSD3B (**Figures 5G, I**) or CYP17A1 (**Figures 5L, M**), while CD51++ macrophages (**Figures 5F, H**) as expected did not. Also, CYP17A1 was not detected for cells without differentiation (**Figure 5J**) or with differentiation but no CYP17A1 primary antibody during immunofluorescent staining (**Figure 5K**). As expected, LCs developed from young CD51+ SLCs (**Figure 5G**) showed higher HSD3B activity than LCs derived from old CD51+ SLCs (**Figure 5I**).

### 3.5 Effects of macrophages on SLC proliferation and differentiation

**Figure 6:**
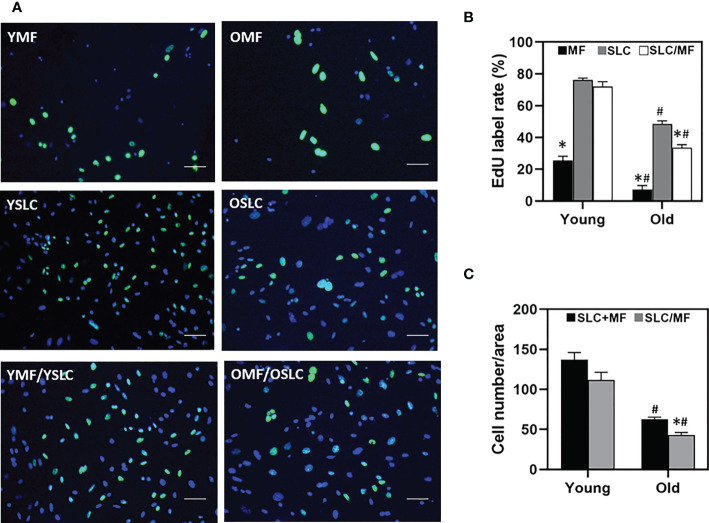
Effects of aging and macrophages on the proliferation of SLCs. **(A)** Dividing cells were labeled with EdU for 6 hours on day 3 in culture. YMF or OMF: Young or old CD51++ macrophages cultured alone. YSLC or OSLC Young or old CD51+ SLCs cultured alone. YMF/YSLC or OMF/OSLC: Young or old CD51++ macrophages were co-cultured with young or old CD51+ SLCs. **(B)** Quantification of EdU labeling indices of CD51+ SLCs cultured separately, CD51++ macrophages (MF) cultured separately, or co-culture of the two cell types (SLC/MF). **(C)** Quantification of the total cell numbers of cells cultured separately (SLC+MF) or co-cultured cells (SLC/MF). The data are expressed as mean ± SEM of three individual experiments. *^,#^Significantly different from age-matched SLC **(B)** or SLC+MF **(C)** controls (*) or from same cell type from young animals (^#^) at P < 0.05 respectively. Scale bars: 50µm in length.

With full confirmation that CD51+ and CD51++ fractions represent SLC and macrophages, respectively, we further examined how macrophages may affect the proliferation and differentiation of CD51+ SLCs by co-culture of the two cell types. Co-culture of CD51++ macrophages and CD51+ SLCs from young animals did not result in a significant change in proliferation of CD51+ SLCs. Co-culture with old cells, however, resulted in a significant reduction in the proliferation of CD51+ SLCs (**Figures 6A, B**). Quantification of the total number of cells accumulated by 4 days confirmed the previous EdU results. The number of old CD51+ SLCs accumulated was about 40% of that with young cells without co-culture with CD51++ macrophages (**Figure 6C**). Co-culture with age-matched CD51++ macrophages reduced the total number of young CD51+ SLCs slightly (by 15%, not significant), while reducing the old cells significantly (by 30%). The results suggest that aging may increase the potential of CD51++ macrophages to inhibit SLC proliferation.

**Figure 7:**
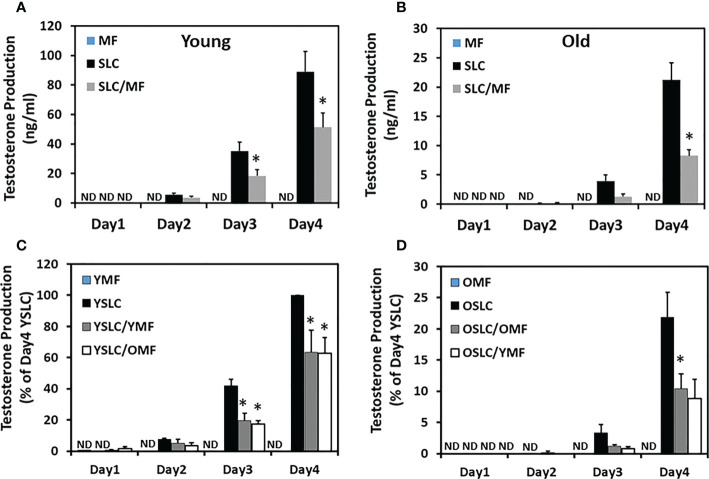
Effect of aging on the interactions between CD51+ SLCs and CD51++ macrophages. **(A, B)** Testosterone production by separately cultured (CD51++ MF or CD51+ SLC) or co-cultured (SLC/MF) cells in the presence of differentiation inducing medium. **(C, D)** Percentage of testosterone production by separately cultured cells (YMF, YSLC, OMF or OSLC) or co-cultured cells within same ages (YSLC/YMF or OSLC/OMF) or across different ages (YSLC/OMF or OSLC/YMF) in the presence of differentiation inducing medium. The data are expressed as mean ± SEM of 3-7 individual experiments. (ND) not detected. *Significantly different from time-matched CD51+ SLC controls at P < 0.05 respectively.

To further examine how macrophages may affect SLC differentiation, testosterone production was compared between individual- and co-cultured cells of both young and old ages (**Figure 7**). During 4 days in culture with differentiation-inducing medium, testosterone production increased with time for both young and old SLCs, while as expected macrophages (MF) never produce any testosterone at any time. Co-culture of the two cell types (SLC/MF) resulted in significant reduction in testosterone production for both ages (by 40% reduction in the young and 60% reduction in the old) (**Figures 7A, B**). Clearly, co-culture of the two cell types resulted in more severe reductions in testosterone production for old cells than for the young.

The more severe reduction in testosterone production by co-culture of old cells with old cells could result from aging of CD51+ SLCs, aging of CD51++ macrophages, or both. To further elucidate the effects, we performed co-culture experiments with the two cell types switched across the two ages, with young SLCs cultured with old macrophages and verse vasa (**Figures 7C, D**). Co-culture of young CD51+ SLCs with CD51++ macrophages of either age resulted in a similar 40% reduction in testosterone production by day 4 (**Figure 7C**), while co-culture of old CD51+ SLCs with CD51++ macrophages of either age resulted in a similar 60% reduction in testosterone production by 4 days (**Figure 7D**). These results suggest that the age-dependent differences between the co-culture experiments were primary determined by the age of the CD51+ SLCs, not the CD51++ macrophages.

Since cell proliferation was also affected by co-culture of CD51+ SLCs with macrophages, the reduction in testosterone productions could be due to either low cell numbers or a reduction in testosterone production by individual cells. To further elucidate the effects, testosterone production was adjusted by cell number determined after the expansion period (**Supplemental Figure S5**). The difference in testosterone production becomes smaller than the unadjusted results. Specifically, the difference induced by macrophages in young cells (about 20% reduction) was no longer statistically significant, while the difference in old cells (about 40%) was still significant, suggesting an aging-related change in the interactions between the two cell types.

### 3.6 Effect of aging on the expression of steroidogenic and macrophages genes

**Figure 8:**
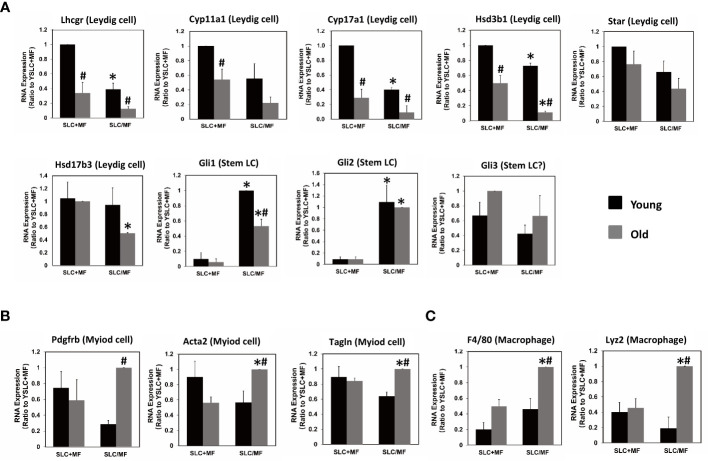
Effects of aging and macrophages on the expressions of LC-, myoid cell- or macrophage-related genes. **(A)** LC-related genes. **(B)** Myoid cell-related genes. **(C)** Macrophage-related genes. (SLC+MF) RNA from the separately cultured cells combined after culture. (SLC/MF) RNA from co-cultured CD51+ SLCs and CD51++ macrophages. The data are expressed as mean ± SEM of three individual experiments. *^,#^Significantly different from age-matched SLC+MF controls (*) or from same cell type from young animals (^#^) at P < 0.05 respectively.

The results so far show that both aging and the presence of CD51++ macrophages can affect CD51+ SLC differentiation. To further elucidate the detailed effects on the steroidogenic pathway, major macrophage and steroidogenic genes were compared by qPCR (**Figure 8**). To rule out a possible dilution of Leydig cell genes by macrophages in co-culture experiments, individually cultured CD51+ SLCs and CD51++ macrophages were combined (SLC+MF) before RNA was extracted. So mRNA levels were compared for the co-cultured cells (SLC/MF) and combined individual cell types (SLC+ MF) within and between the two ages (**Figure 8**). Interestingly, not all steroidogenic genes were inhibited by the co-culture at a same percentage. The key steroidogenic genes Lhcgr, Cyp11a1, and Cyp17a1 were inhibited by co-culture at similar or even larger percentages as compared to testosterone production (**Figures 7A, B**), while other steroidogenic genes Hsd3b1, Star and Hsd17b3 were inhibited much less or not at all by CD51++ macrophages, suggesting that both aging and the presense of macrophages affected CD51+ SLC differentiation by targeting specific genes, not by affecting the overall commitment of SLCs to an LC lineage. In contrast to the inhibition of LC steroidogenic genes, the expression of SLC-associated DHH-signaling genes Gli1 and Gli2, but not Gli3, were up-regulated very significantly by co-culture with CD51++ macrophages for both ages, indicating that macrophages prevented the down-regulation in CD51+ SLC-associated genes that would be otherwise reduced during differentiation to LCs.

Since SLCs have the potential to form both LCs and myoid cell lineages *in vitro* (20, 28) and *in vivo* (20, 29), we also examined whether aging or macrophages may differentially affect CD51+ SLCs’ potential to form one lineage over the other. Clearly aging did not affect the expression of most myoid markers, such as Pdgfrb, Acta2 and Tagln when the cells were cultured separately (SLC+MF) (**Figure 8B**). However, co-culture with CD51++ macrophages (SLC/MF) resulted in specific increases of the three genes in old but not young cells, suggesting that aged CD51+ SLCs may have increased potential to form myoid lineages and reduced potential to form LCs, especially in the presence of macrophages. This has significance for maintaining reproductive capacity with age.

In co-culture experiments, cell interactions could go both ways. To examine whether CD51+ SLCs may also affect CD51++ macrophages, we assayed the expressions of two typical macrophage specific genes, Adgre1 (F4/80) and Lyz2 (**Figure 8C**). Interestingly, aging and CD51+ SLC treatment synergistically up-regulated the two genes, suggesting that SLCs may also affect macrophage genes.

## 4 Discussion

### 4.1 Characterization of CD51+ and CD51++ cells

SLCs have been identified and/or isolated from adult rodent and human testes based on different markers (6, 12, 15–18, 30). CD51 was identified aa a particularly useful marker to isolate SLCs from mouse testes (12, 15). However, our earlier work showed that flow-cytometry profiles of testicular cell suspensions suggested that the adult mouse testes contained two different CD51 populations: a weakly-positive population (CD51+) also expressing SLC markers and a strongly-positive (CD51++) population expressing macrophage markers (16). In the present study, we further characterized these two CD51 positive populations both *in vitro* and *in vivo* with more specific protein markers and confirmed their identities with added certainty.

The current study clearly demonstrated two CD51-positive populations and their cell types, CD51+ as SLCs and CD++ as macrophages, based on 4 lines of evidence: First, whole-mounted seminiferous tubules and unsorted testicular cell suspensions were co-stained with macrophage marker F4/80. Only part of CD51-positive cell population co-stained with F4/80 antibody, suggesting F4/80-positive cells were included in the whole CD51-positive population. Moreover, seminiferous tubule CD51-positive cells co-stained by F4/80 showed an elongated shape with multiple branches, typical morphology for macrophages found along the tubule surface of adult mice (31). Second, part of CD51-positive cell population was capable of accumulating trypan blue granules in their cytoplasm *in vivo*, a well-established property of macrophage phagocytosis (26, 27). Third, CD51++ cells expressed all the well-known macrophage marker genes, while CD51+ cells only expressed major SLC marker genes. Neither cell type expressed any of the markers for other major testicular cells. Fourth, FACS sorted CD51+ cells could be induced to differentiate into Leydig cells while CD51++ cells could not.

Although the CD51++ faction expressed high levels of macrophage markers, the purity of the population was not further examined in the current study. The possibility that it may contain other cells, especially leukocytes cannot be ruled out. However, since macrophages are by far the most predominated immune cell type in the interstitial compartment, the minor contamination from other cells, such as leukocytes, should not affect the final results and conclusion significantly. Nevertheless, further studies are required to characterize the CD51++ population in depth in order to fully establish its macrophage identity.

Multiple SLC markers have been reported for stem Leydig cells from various species. We compared the expression of SLC markers and markers for general mesenchymal stem cells, since previous studies suggested that SLCs may belong to the general mesenchymal stem cell population (20, 32). Consistent with previous findings, CD51+ but not CD51++ cells specifically expressed SLC markers Nes, Coup-tf2, Tcf21, and Pdgfra, but failed to express SLC markers Cd90 and P75ntr. The failure to express the last two markers may reflect a species difference, since Cd90 was identified in rat (18), while P75ntr was established with human cells (17). A recent scRNA-seq study found that in mouse testis Cd90 was specifically expressed by lymphocytes, instead of SLCs, suggesting its species-limited specificity (33). In addition to SLC markers, CD51+ cells also expressed major mesenchymal stem cell markers Cd105, Cd73, and Cd44 and did not express the negative markers Cd45 and Cd31. Overall, CD51+ cells expressed the most markers for both SLC and general mesenchymal stem cells, supporting their designation as SLCs.

### 4.2 Effects of macrophages on SLC proliferation and differentiation

Previous studies suggested that LC development critically depended on macrophages *in vivo* (34). By simultaneously isolating and sorting SLCs and macrophages with the CD51 marker from the same samples, we could more fully study how macrophages might affect SLC proliferation and differentiation using co-culture of the two cell populations *in vitro*. The results consistently showed that CD51++ macrophages had inhibitory effects on both the proliferation and differentiation of CD51+ SLCs. These results were a little surprising given a previous observation that the macrophages were required for the development of LCs *in vivo* (34). However, consistent with the current study, more recent *in vitro* studies showed that SLCs can be induced to differentiate into Leydig cells in the absence of macrophages, questioning whether macrophages are really essential for LC development (6, 7, 10, 12, 14, 17, 18, 30). The reason behind the differences between *in vivo* and *in vitro* studies is still unknown. Two possibilities may explain the inconsistency. First, the *in vivo* study eliminated macrophages by intratesticular injection of dichloromethylene diphosphonate-containing liposomes, which could be toxic to SLCs and/or paracrine producing cells. Another possibility could be that macrophages function differently *in vivo* and *in vitro*. Further studies are required to establish the regulatory roles of macrophages on LC development *in vivo*.

### 4.3 Effects of aging on SLC gene expression and function

Most of the SLC marker genes examined increased with age, including Cd51, Nes, Coup-tf2, Cd14, Cd44, Cd73, Cd34, Des, and Pdgfrb, while Tcf21, Pdgfra, Cd105, Notch2, Hes1, and Smo did not. Interestingly, all the inflammatory-related cytokine genes examined, including Tnfa, IL-1b, IL-6, and IL-8, were dramatically up-regulated with age in both CD51+ SLCs and CD51++macrophages, suggesting that aging may increase inflammatory stress in mouse testes.

Compared to the young cells, old CD51+ SLCs reduced their proliferation activity by 30% and differentiation by almost 2-fold. Our work suggests three changes in SLCs may explain these effects. First, the expression of Ptch1 decreased significantly with age, suggesting a reduction in response to DHH, a SLC differentiation inducing molecule (18). Second, the expression of Notch-2 and -3 were up-regulated, indicating an increase in Notch signaling, a negative regulator of SLC differentiation (35). Third, expression of multiple inflammatory cytokine genes by SLCs were up-regulated by age, suggesting an increase in autocrine inhibition by cytokines. The detail mechanisms by which SLCs age need further studies.

### 4.4 Effects of aging on macrophage phenotypes and functions

Genes associated with macrophage surface proteins, such as Adgre1 and Cd115 (Csf1r) were decreased with age, while genes associated with intracellular or secretory proteins, such as Lyz2, C1qb, IL-1b and IL-8 were all up-regulated with age, suggesting that old testicular macrophages adopted proinflammatory characteristics. Aging not only altered the gene expression profiles of macrophages but also their function. Since co-cultures of SLCs and macrophages reduced cell division, the differences induced by macrophages in the co-culture experiment may represent a combination of changes in both cell numbers and differentiation of cells. Adjustment of testosterone production by cell number suggests that macrophages affected SLC proliferation and differentiation at similar levels within each matched age co-cultures (20% each for young cells and 30% each for the old), so the final Leydig cell development (proliferation plus differentiation) were reduced by 40% for the young and 60% for the old. These results suggest that macrophages can inhibit LC development by affecting both the proliferation and differentiation of SLCs, and these effects were enhanced with aging.

### 4.5 Does the aging affect SLC lineage preference?

It is well known that LCs and myoid cells develop from the same progenitor pool in fetal, pubertal, and adult testes (20, 28, 29, 36). A possible competition may occur between the two lineages for the same progenitor pool. Does the age-related reduction in LC differentiation relate to an increase in SLCs’ ability to form myoid lineages? The results support such an hypothesis. First, old SLCs expressed significantly higher levels of genes related to myoid cell differentiation, such as Des and Pdgfrb. Moreover, in the presence of macrophages old SLCs had not only reduced ability to form LCs, but also increased expression of the major myoid markers, including Acta2, Tagln, and Pdgfrb. These results strongly suggest that aging may affect the lineage preference of SLC from LCs to myoid cells. Whether such a phenomenon happens *in vivo* deserves further study.

In (**Figure S6**) summary, mouse SLCs and macrophages can be isolated from a single testicular cell suspension by FACS, based on their differential expression of the CD51 protein. Aging increased inflammatory-related cytokine gene expression in both CD51+ SLCs and CD51++ macrophages. CD51+ SLCs from old testes had reduced capacity to proliferate and differentiate into LCs, with a more significant reduction in differentiation than in proliferation. In addition, CD51++ macrophages can inhibit CD51+ SLC development, with a more significant reduction in old (60%) than young (40%) cells. Overall, the study suggests that aging and macrophages can inhibit LC development by affecting both SLC proliferation and differentiation. More studies are required to elucidate the molecular mechanisms underneath the changes.

## Data availability statement

The original contributions presented in the study are publicly available. This data can be found here: https://figshare.com/s/b300079f988a8914b2bf.

## Ethics statement

The animal study was reviewed and approved by Wenzhou Medical University Animal Care and Use Committee.

## Author contributions

JS and JW: Conceptualization, Investigation, Methodology, Formal analysis, Data curation, Visualization and Writing. XW, JX, FH, XG, and XH: Methodology, Data curation and Validation. PD, HC, and CC: Conceptualization, Supervision, Project administration, Funding acquisition and Writing. All authors contributed to the article and approved the submitted version.
