# Supplementary material for: Effects of aging and macrophages on mice stem Leydig cell proliferation and differentiation in vitro
Source: Front Endocrinol (Lausanne). 2023 Mar 27;14:1139281. doi: 10.3389/fendo.2023.1139281 (PMC10083278; doi:10.3389/fendo.2023.1139281)
Supplement: Supplementary file 1 [file DataSheet_1.pdf]

Supplementary Materials for Shao J et al., Effects of aging and macrophages on stem Leydig cells proliferation and differentiation in vitro

Supplementary Table S1: Antibodies information

Primary Antibody name	Dilutions	Vendor name	Cat.No.
CD51-PE	1:40	eBioscience, San Diego, CA	2087658
F4/80-APC	1:20	MultiSciences, Hangzhou, CHN	70-AM048005-100
CYP17A1	1:200	Cell Signaling Technology, MA, Boston, USA	94004
HSD3B1	1:500	Novus Biologicals,Littleton, Colorado, USA	NB110-78644
CD51	1:500	Abcam, Cambridge, UK	ab179475
F4/80	1:500	Abcam, Cambridge, UK	ab16911
Dylight 488 conjugated of goat anti-Rat IgG (H+L)	IF(1:400)	MultiSciences, Hangzhou, CHN	060702
Dylight 594 conjugated of goat anti-Rabbit IgG (H+L)	IF(1:400)	MultiSciences, Hangzhou, CHN	A00743
Goat anti-Rabbit IgG-AlexaFlour 488	IF(1:500)	Absin,Shanghai,CHN	abs20025

Supplementary Table S2: Primers information

Gene name	Forward sequence(5'to3')	Reverse sequence(5'to3')
Cyp17a1	ATCTTGGCTTGTATCAGAATG	ACTTGGAAATGATAAAGGAAC
Scarb1	AGCGGGGTGTAGGGACTGGGT	GTTCTGCCGTTGCTGTGGTTC
Acta2	TGAGACCTTCAATGTCCCCGC	TCACACCATCTCCAGAGTCCAGC
CD146	ACAGCCACGATGACCACA	ATACCTGACTCCAGCCAAAC
CD31	AGCACCGAAGTACCATTT	CAGATAAGCCCACCAGAG
DDX4	TAAAAGGGTTTGGCGTTGTTC	CCAGTTTGGTCATTCAGTTCG
nestin	CCTCAACCCTCACCACTCTATTT	TCCAGACCACTTTCTTGTATTTCCT
Coup-TF2	ACCTACCAAACGGACGAAAA	TGCCTGTGGTCTGTCTGATG
PDGFRa	ACTCGCTGGTCTTGAACG	CTGGTGCCTGCCTCCTAT
ADGRE1	TCGATGTCTAGGTACTCCGTC	CTGTGGAAAGCACCATGTTAG
CD115	CAGGGTCCAAGGTCCAGTAGG	TGGTTGTAGAGCCGGGTGAAA
TNF-a	CTTGTTGCCTCCTCTTTTGCTTA	CTTTATTTCTCTCAATGACCCGTAG
IL-6	TCACAGAAGGAGTGGCTAAGGACC	ACGCACTAGGTTTGCCGAGTAGAT
IL-8	TGTTCACAGGTGACTGCTCC	AGCCCATAGTGGAGTGGGAT
IL-1b	TGTGTTTTCCTCCTTGCCTCTGAT	TGCTGCCTAATGTCCCCTTGAAT
Lhcgr	TTAGCCAAATCAACACCCTAA	GTTCACCCAAGACACTCCAAT
Star	TGAGTGATGACCGTGTCTTTT	GGGACGAAGTGCTAAGTAAGA
Cyp11a1<br>Hsd3b1	GAAGTCTGGAGGCAGGTTGAG<br>TTTTCTGCTTTGCTTCCTCCC	ACCTATTCCGCTTTTCCTTTG<br>CCTTCCTCTGCCCCTGCTCTA
Hsd17b3	TGAGCAAGGCAGCCACAGGAT	GATGACCAAGACCGCCGATGA
Gli1	GACTTTCTGGTCTGCCCTTTT	AGCCCGCTTCTTTGTTAATTTGA
Gli2	GGGACTCTTTAGCCTCGCAG	CCACAGGGTTGAGGTAGTCAT
Gli3	GAAGAAACGCAATCACTATGCAG	GTCCCACGGTAAGGGAGAGA
PDGFRb	AGGCTTGCTTCTCGCTAC	ATCTACGTGGACCCTGTGC
Tagln	CCAACAAGGGTCCATCCTACG	ATCTGGGCGGCCTACATCA
Lyz2	ATGGAATGGCTGGCTACTATGG	ACCAGTATCGGCTATTGATCTGA
Lipe	GACTATGGGTGACGTGTAGAG	AAGCCAAAGATGAAGTGAGAC
CD45	GTTTTCGCTACATGACTGCACA	AGGTTGTCCAACTGACATCTTTC
CD51	CGGGTCCCGAGGGAAGTTA	TGGATGAGCATTCACATTTGAGA
TCF-21	CTCCCTGAAAGTGGACTCCAA	CGGGCTTTTCTTAGTGGGC
CD105	AGGGGTGAGGTGACGTTTAC	GTGCCATTTTGCTTGGATGC
CD73	CCTGCACACAAACGACGTG	CTGGTCTCCGGCATCCAAAA
Notch1	CCCTTGCTCTGCCTAACGC	GGAGTCCTGGCATCGTTGG
Notch2	GAGAAAAACCGCTGTCAGAATGG	GGTGGAGTATTGGCAGTCCTC
Notch3	AGTGCCGATCTGGTACAACTT	CACTACGGGGTTCTCACACA
Hes1	TCAACACGACACCGGACAAAC	ATGCCGGGAGCTATCTTTCTT
Ptch1	GCCTTCGCTGTGGGATTAAAG	CTTCTCCTATCTTCTGACGGGT
Ptch2	GGTCCTCCGCACCTCATATC	GTCTGTCTCAATTACAGCCACTC
Smo	GTGCTGTCTACATGCCCAAGT	GCAACGCAGAAAGTCAGGC
Sufu	CGGACCCCTTGGACTATGTTA	CTTCAGACGAAACGTCAACTCA
Desmin	CAATCTCGCAGGTGTAGGA	ACTCAGGCAGCCAATAAGA

CD44	AGAAAAATGGCCGCTACAGTATC	TGCATGTTTCAAAACCCTTGC
CD14	CTCTGTCCTTAAAGCGGCTTAC	GTTGCGGAGGTTCAAGATGTT
CD34	CTGGGTAGCTCTCTGCCTGAT	TGGTAGGAACTGATGGGGATATT
CD90	GCTAGGGTAAGGACCTTGATAT	GCCGCCATGAGAATAACA
P75NTR	CAACCACAGCAGCCAAGAT	GCCGATACGGTGACCACT
Ccnd1	TGACTGCCGAGAAGTTGTGC	CTCATCCGCCTCTGGCATT
IL-1a	ATCCAAACTGTCCCTCCA	GGGGCTTTATCATCCTCA
INSL3	GGCTAGAGCAGAGACATC	GGACACAGACCCAACAGG
C1qb	CGTCGGCCCTAAGGGTACT	GGGGCTGTTGATGGTCCTC
Rgs5	GGGTTGCCTGTGAGAATTACA	TGAAGTGGTCAATGTTCACCTCT
Rps16	TTTGAGATGGACTGTCGGATG	AAGTTACTGGAGCCTGTTTTG

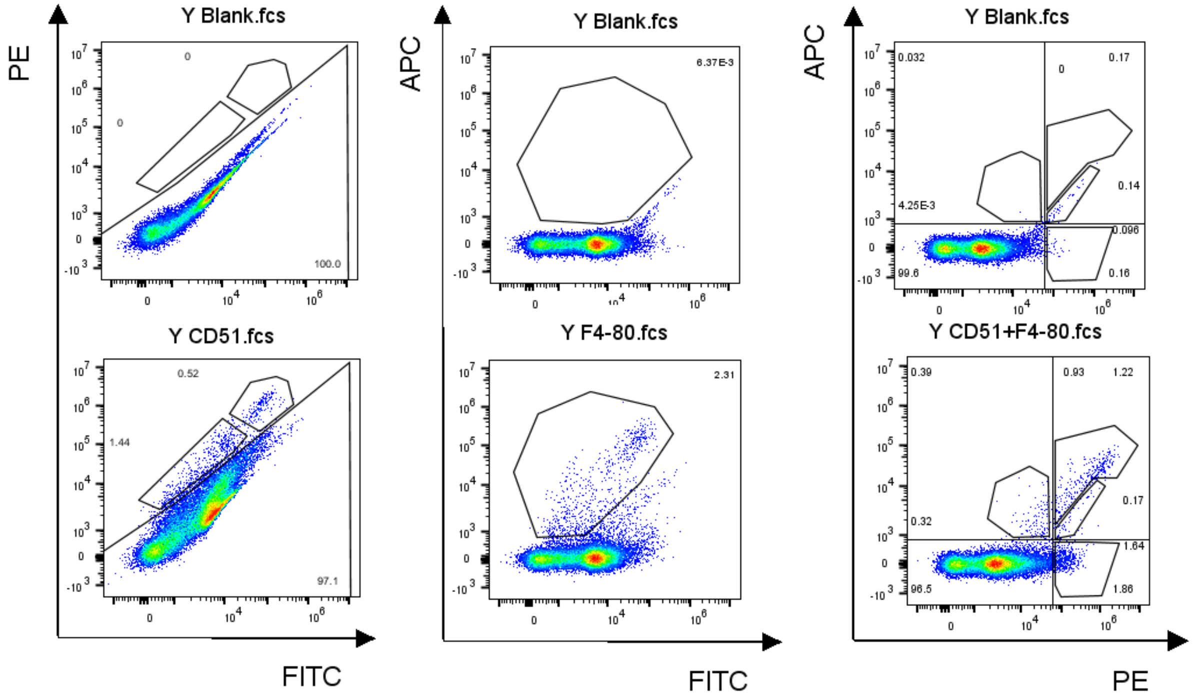

Supplementary Fig S1: Flow cytometry analysis of testicular cells of young adult mouse co-stained with CD51-PE and F4/80-APC antibodies. Top: Unstained cells; Bottom: Cells co-stained with CD51 and F4/80-APC antibodies. Cells were displayed by PE/FITC (left panel), APC/FITC (middle panel) or PE/APC (right-panel) channels. The bottom-right diagram shows that the cells with co-staining of the two antibodies represent 1.93% of the total cell population.

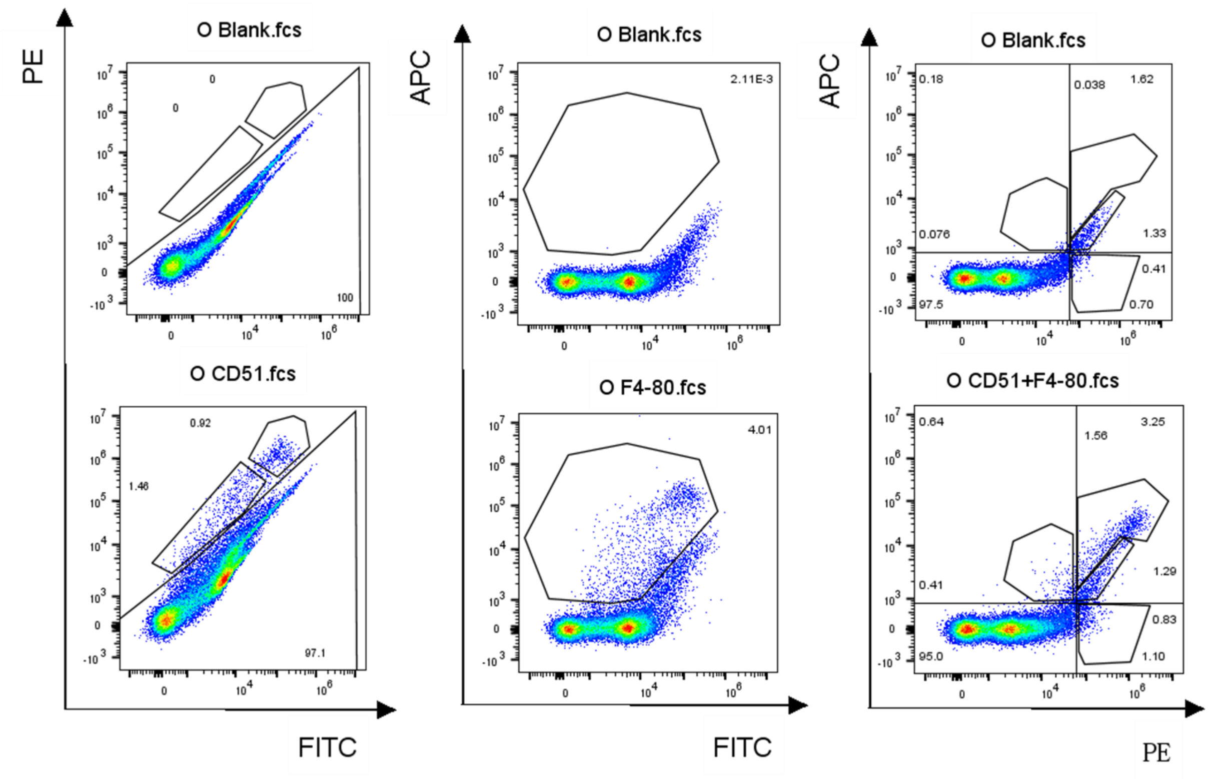

Supplementary Fig S2: Flow cytometry analysis of testicular cells of old mouse co-stained with CD51-PE and F4/80-APC antibodies. Top: Unstained cells; Bottom: Cells co-stained with CD51 and F4/80-APC antibodies. Cells were displayed by PE/FITC (left panel), APC/FITC (middle panel) or PE/APC (right-panel) channels. The bottom-right diagram shows that the cells with co-staining of the two antibodies represent 3.01% of the total cell population.

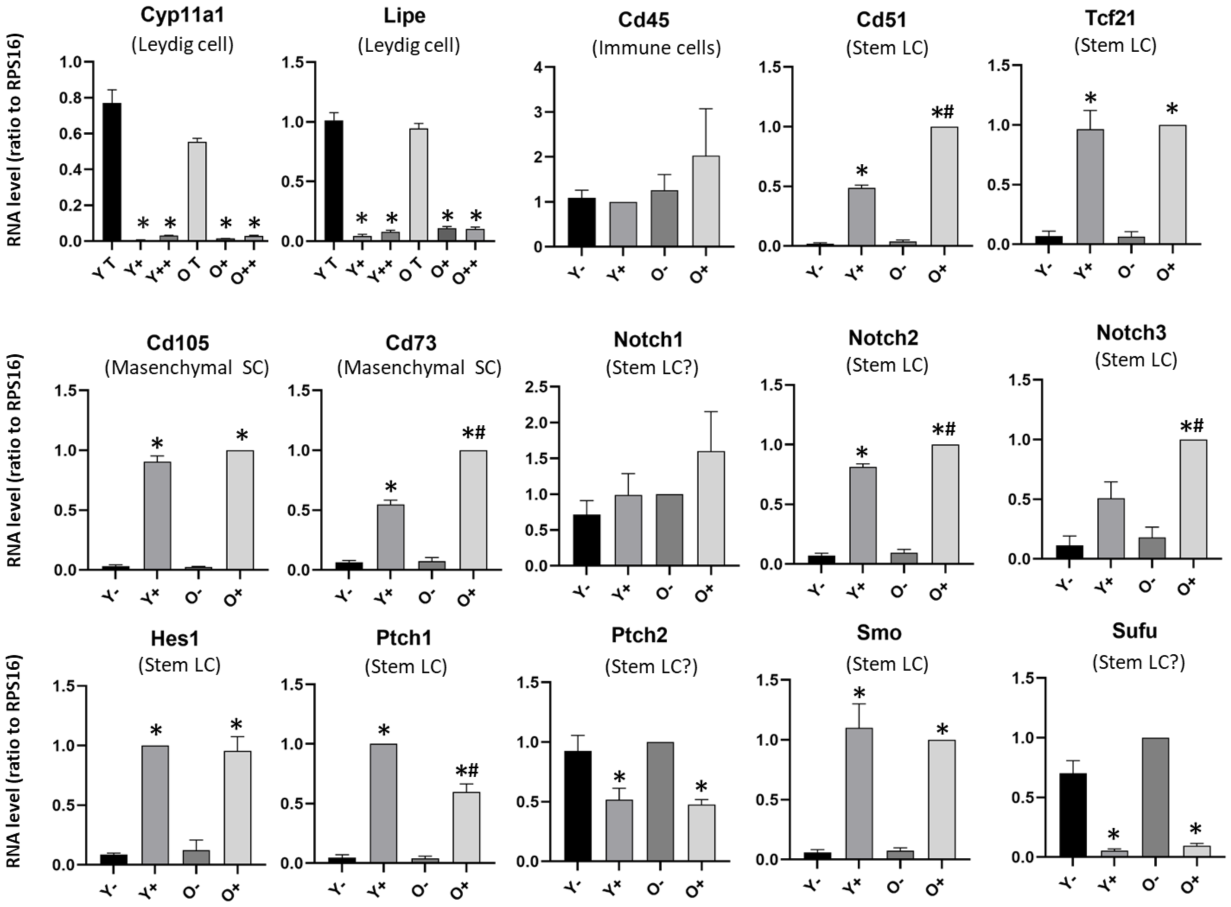

Supplementary Fig S3. Expressions of testicular cell marker genes by the isolated CD51 positive cells of the young and old testes. RNAs from whole testis (YT or OT) or CD51-negitive cells (Y- or O-) were used as controls. Genes include Cyp11a1 and Lipe (Leydig cells), CD45 (immune cells), all others (stem Leydig cell, Stem LC). The data is expressed as mean ± SEM of cells from three individual experiments. *,#Significantly different from the age-matched YT/OT or Y-/O- controls (*) or from same cell types of young animals (#) at $P < 0.05$ respectively.

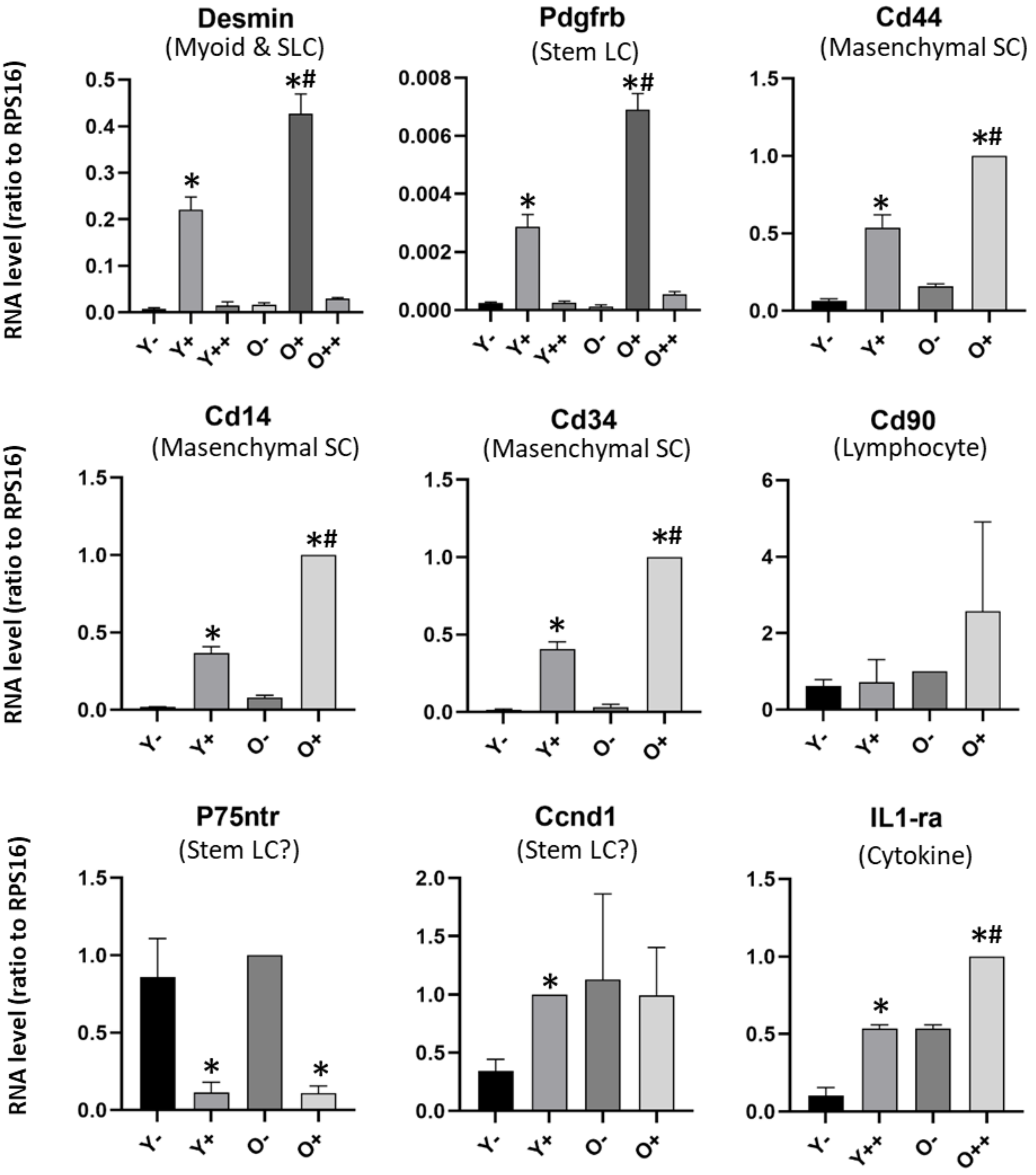

Supplementary Fig S4. Expressions of marker genes of stem Leydig cells (Stem LC, SLC) or general mesenchymal stem cells (Mesenchymal SC) by the isolated CD51 positive cells of the young and old testes. RNAs from CD51-negitive (Y- or O-), weakly positive (Y+ or O+) and strongly positive (Y++ or O++) cells were analyzed. SC: stem cell. The data is expressed as mean ± SEM of cells from three individual experiments. *,#Significantly different from the age-matched Y-/O- cells (*) or from same cell types of young animals (#) at $P < 0.05$ respectively.

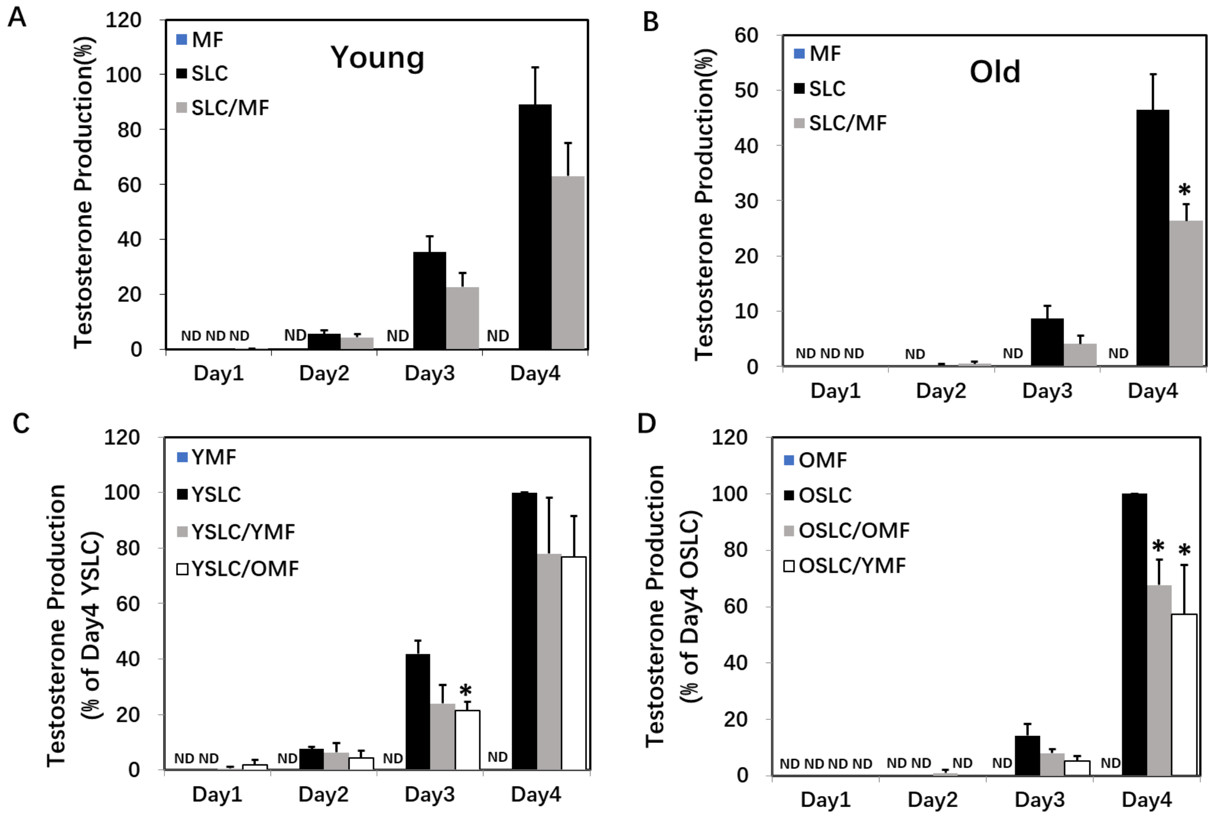

Supplementary Fig S5. Effect of aging on the interactions between SLCs and macrophages. (A, B) Testosterone production by individually cultured (MF or SLC) or co-cultured (SLC/MF) cells in the presence of differentiating inducing medium. (C, D) Percentage of testosterone productions by individually cultured cells (YMF, YSLC, OMF or OSLC) or co-cultured cells within same age (YSLC/YMF or OSLC/OMF) or across different ages (YSLC/OMF or OSLC/YMF) in the presence of differentiating inducing medium. The data were derived from Figure 7 and were normalization by cell numbers. Data are expressed as mean ± SEM of 3-7 individual experiments. (ND) not detected. *Significantly different from the time-matched SLC controls at $P < 0.05$ respectively.

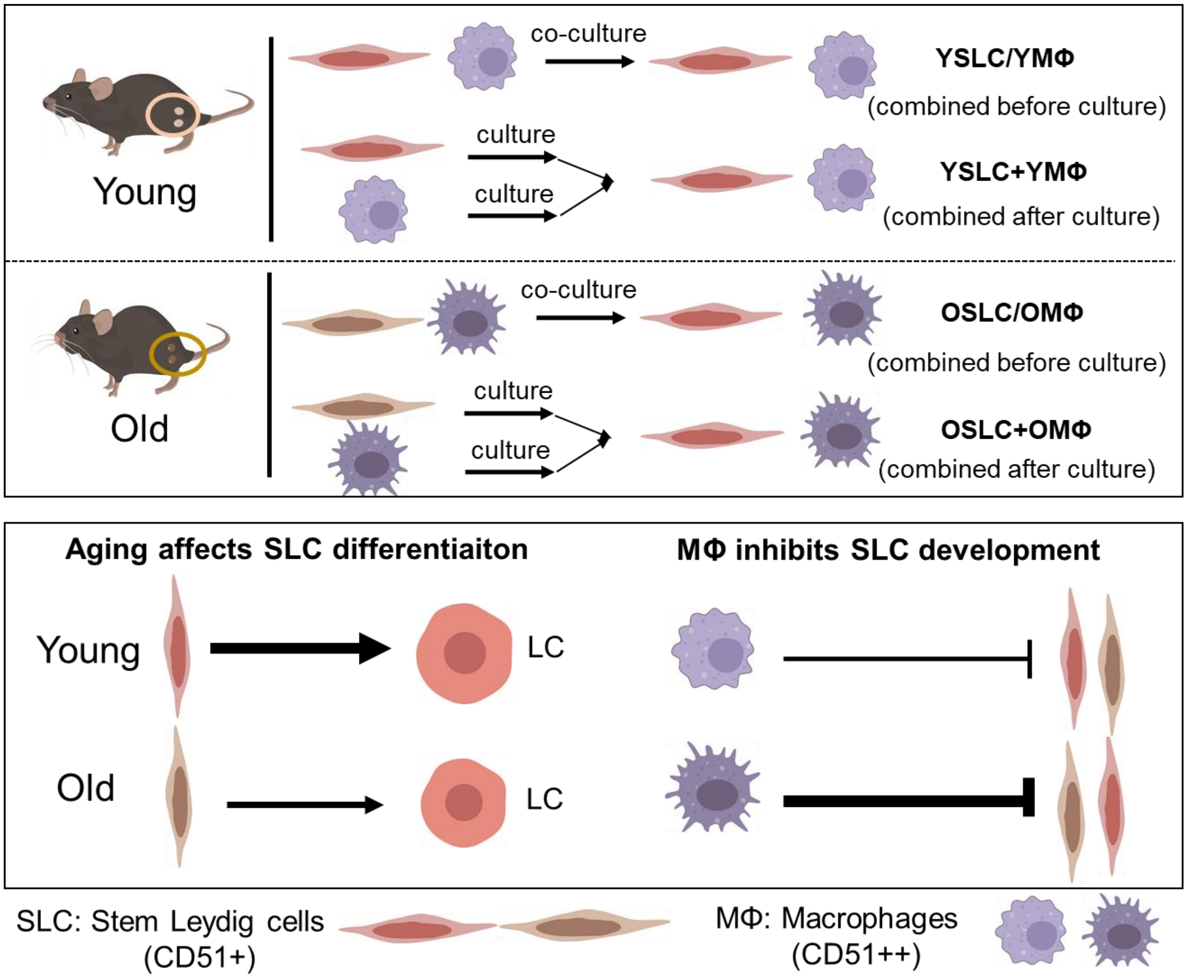

Supplementary Fig S6. Graphic summary of the study design and results.
